# Supplementary material for: Cohort profile: The Chikwawa lung health cohort; a population-based observational non-communicable respiratory disease study of adults in Malawi
Source: PLoS One. 2020 Nov 12;15(11):e0242226. doi: 10.1371/journal.pone.0242226 (PMC7660567; doi:10.1371/journal.pone.0242226)
Supplement: S1 File — (DOCX) [file pone.0242226.s001.docx]

# **Link to minimal anonymized dataset**

Accessible through Mendeley

Link: <https://data.mendeley.com/datasets/whbk485wbw/1>

DOI: <http://dx.doi.org/10.17632/whbk485wbw.1>
