# Supplementary material for: Cohort profile: The Chikwawa lung health cohort; a population-based observational non-communicable respiratory disease study of adults in Malawi
Source: PLoS One. 2020 Nov 12;15(11):e0242226. doi: 10.1371/journal.pone.0242226 (PMC7660567; doi:10.1371/journal.pone.0242226)
Supplement: S2 File — (PDF) [file pone.0242226.s002.pdf]

Table of Contents

1 Appendices: Data collection tools in English and Chichewa..2

1.1 Appendix A: Spirometry questionnaire 2

1.2 Appendix B: Core questionnaire 4

1.3 Appendix C: Verbal autopsy questionnaire 15

1.4 Appendix D: Chichewa translation of the Verbal autopsy questionnaire. 20

1.5 Appendix E: Chichewa translation of the Core questionnaire. 25

1.6 Appendix F: Chichewa translation of the Spirometry questionnaire. 48

1 Appendices: Data collection tools in English and Chichewa

1.1 Appendix A: Spirometry questionnaire

Identification
Site
Fieldworker number
Participant number
1. Pulse (bpm)
2.1. Systolic blood pressure (mmHg)
2.2. Diastolic blood pressure (mmHg)
3. Height (cm)
4. Weight (kg)
5.1. Hip circumference 1st measurement (cm)
5.2. Hip circumference 2nd measurement (cm)
6.1. Waist circumference 1st measurement (cm)
6.2. Waist circumference 2nd measurement (cm)
7. Ulna length (cm)
8. Fibula length (cm)
9.1. Neck circumference 1st measurement (cm)
9.2. Neck circumference 2nd measurement (cm)
10. Comments
11. In the past three months have you had any surgery on your chest or abdomen?
12. Have you had a heart attack within the past three months?
13. Do you have a detached retina or have you had eye surgery within the past three months?
14. Have you been hospitalized for any other heart problem within the past month?
15. Are you in the last trimester of pregnancy?
16. Are you currently taking medication for tuberculosis?
17. Is there some other reason why this participant should not perform the spirometry maneuver?
DO NOT PERFORM SPIROMETRY!
18. Have you had a respiratory infection (cold) in the last three weeks?
19. Have you taken any medications for breathing in the last 24 hours?
**Recent medications**
20. Record name/type of medication(s) used:
20.1. Did participant use a short acting beta agonist (e.g. albuterol, salbutamol) or anticholinergic inhaler (e.g. atrovent, iprtropium), either alone or in combination with some other product, in the last six hours?
20.2. Did participant use a long acting beta agonist (e.g.Serevent, Advair, Formoterol, Symbicort) or oral beta 2 agonist (e.g. salbutamol tablets), either alone or in combination with some other product, in the last 12 hours?
20.3. Did participant use an oral theophyllin/ long acting anticholinergic (e.g. spiriva, tiotropium), either alone or in combination with some other product, in the last 24 hours?

21. Have you smoked in the last 30 days?
21.1. When did you last smoke?
22.1. Acceptable pre-bronchodilator test completed?
22.2. Acceptable post-bronchodilator test completed?
23. Why were you unable to obtain satisfactory spirometry?
24. Were any adverse events related to the spirometry maneuver observed by the evaluator?
25. Was this a major event (was the participant hospitalised or did the participant die)?
26. Please briefly describe event:
27. If the participant had a condition that would affect the result of their spirometry test (e.g., kyphosis, missing limbs, etc.) note that condition here.

1.2 Appendix B: Core questionnaire

**Identification**
Site
Fieldworker number
Participant number
1. What is the participant's sex?
2. What is your date of birth?
**Education**
3. What is the _highest level_ of schooling your _mother_ has completed?
**Household assets**
4.1. Please tell me whether this household or any person who lives in the household has/owns the following items:
4.1.1. Electricity?
4.1.2. Flush toilet?
4.1.3. Fixed telephone?
4.1.4. Cell telephone?
4.1.5. Television?
4.1.6. Radio?
4.1.7. Refrigerator?
4.1.8. Car?
4.1.9. Moped/scooter/motorcycle?
4.1.10. Washing machine?
4.1.11. Own their own home?
4.1.12. Indoor bath or shower?
4.1.13. Indoor tap?
4.1.14. Outdoor tap of their own?
4.1.15. Country to specify
4.1.16. Country to specify
4.2. When you were _5 years old_ did any person who lived in your household have/own the following items:
4.2.1. Electricity?
4.2.2. Flush toilet?
4.2.3. Fixed telephone?
4.2.4. Television?
4.2.5. Radio?

4.2.6. Refrigerator?
4.2.7. Car?
4.2.8. Moped/scooter/motorcycle?
4.2.9. Washing machine?
4.2.10. Own their own home?
4.2.11. Indoor bath or shower?
4.2.12. Indoor tap?
4.2.13. Outdoor tap of their own?
5. In the last year did you or any person who lives in the household ever go hungry for lack of money?
5.1. How often did you or any person who lives in the household ever go hungry for lack of money?
6. How many _people_ live in your house with you? (including you)
7. How many _rooms_ are there in your house? (excluding kitchen and bathroom/s)
**Respiratory symptoms**
These questions pertain mainly to your __chest__. Please answer yes or no if possible. If you are in doubt about whether your answer is yes or no, please answer no.
8. Do you _usually_ cough when you don't have a cold?
8.1. Are there _months_ in which you cough on _most days_?
8.2. Do you cough on _most days_ for as much as _three months each year_?
8.3. For how _many years_ have you had this cough?
9. Do you _usually_ bring up _phlegm_ from your _chest_, or do you usually have phlegm in your chest that is difficult to bring up when you don't have a cold?
9.1. Are there _months_ in which you have this phlegm on most days?
9.2. Do you bring up this phlegm on _most days_ for as much as three months each year?
9.3. For how many _years_ have you had this phlegm?
9.4. Is the phlegm worse when you lie in certain positions (on one side or the other)?
10. Have you had _wheezing_ or _whistling_ in your chest at any time in the _last 12 months_?
10.1. In the _last 12 months_, have you had this wheezing or whistling _only_ when you have a cold?
10.2. In the _last 12 months_, have you ever had an attack of wheezing or whistling that has made you feel _short of breath_?
11. Are you _unable_ to walk due to a condition _other than_ shortness of breath?
11.1. Nature of condition(s):
Exertional Dyspnoea
12. Are you troubled by shortness of breath when _hurrying on the level_ or _walking up a slight hill_?
12.1. Do you have to walk slower than people of _your age_ on _level ground_because of shortness of breath?
12.2. Do you ever have to _stop for breath_ when walking at your _own pace_ on _level ground_?
12.3. Do you ever have to stop for breath after _walking about 100 yards_ (or after a few minutes) on _level ground_?

12.4. Are you too short of breath to leave the house or short of breath on dressing or undressing?
**Respiratory diagnoses**
13. Has a _doctor or other health care provider_ ever told you that you have emphysema?
14. Has a _doctor or other health care provider_ ever told you that you have asthma, asthmatic bronchitis or allergic bronchitis?
14.1. Do you _still_ have asthma, asthmatic bronchitis or allergic bronchitis?
15. Has a _doctor or other health care provider_ ever told you that you have chronic bronchitis?
15.1. Do you _still_ have chronic bronchitis?
16. Has a _doctor or other health care provider_ ever told you that you have chronic obstructive pulmonary disease (COPD)?
17. In the past _12 months_, have you taken any medications for your breathing (including medications for nasal congestion)?
**Medications**
17.1. How many medicines have you taken for your breathing (including medications for nasal congestion) in the past 12 months?
Medications: Details
17.2. Medication Name(s):
17.2.1. Formulation:
17.2.2. Is the medicine taken on most days, or just when you have symptoms, or both?
17.2.3. When you are taking the medication, how many _days_ a week do you take it?
17.2.4. When you are taking the medication, how many _months_ in the past 12 months have you taken it?
17.3. Please tell me about any other products that you take or things you do to _help_ your breathing that you have not already told me about.
**Spirometry and breathing problems**
18. Has a _doctor or other health care provider_ ever had you blow into a machine or device in order to measure your lungs (i.e., a spirometer or peakflow meter)?
18.1. Have you used such a machine in the past _12 months_?
19. Have you ever had a period when you had breathing problems that got so bad that they interfered with your usual daily activities or caused you to miss work?
19.1. How many such episodes have you had in the past 12 months?
19.2. For how many of these episodes did you need to see a doctor or other health care provider in the past 12 months?
19.3. For how many of these episodes were you hospitalized overnight in the past 12 months?

19.3.1. All together, for how many total _days_ were you hospitalized overnight for breathing problems in the past 12 months?
**Sleep**
20. How many hours of sleep do you estimate that you get on average each night?
21. Do you snore?
21.1. Your snoring is:
21.2. How often do you snore?
21.3. Has your snoring ever bothered other people?
22. Has anyone noticed that you quit breathing during your sleep?
23. Do you gasp for air or choke while sleeping?
23.1. In the last month on how many nights per week did you gasp for air or choke while sleeping?
24. In the past month, how often have you felt _sleepy during the day_?
25.1. How likely are you to doze off while sitting in a _public place_ (for instance in a theatre or meeting)?
25.2. How likely are you to doze off while sitting down and _talking to someone_?
25.3. How likely were you to doze off while sitting _quietly_ after a meal without alcohol?
26. In the past month, how often have you had _heartburn after lying down_?
27. In the past month, how often have you _sweated or perspired excessively_ during the night?
28. During your waking time, how often do you feel tired, fatigued or not up to par?
29. In the past three months, how often have you woken up at least 30 minutes earlier in the morning and been unable to get back to sleep?
30. In the past three months, how often have you woken from sleep _several_ times during the night?
31. In the past three months, how often have you had difficulties _falling asleep_ (taken more than 30 minutes)?
**Smoking**
31. Now I am going to ask you about smoking. First I will ask about cigarettes, including hand rolled cigarettes, and then I will ask about other items that are smoked.
31.1. Have you _ever_ smoked __cigarettes__?
31.1.1. How _old_ were you when you first started regular cigarette smoking?
31.1.2. Have you _stopped_ smoking?
31.1.2.1. How _old_ were you when you last _stopped_?
31.1.3. On _average_ over the entire time that you smoke(d), how many cigarettes per day/week do (did) you smoke?
31.1.3.1. cigarettes/day
31.1.3.2. cigarettes/week

31.1.4. On average over the entire time that you smoke(d), do (did) you primarily smoke manufactured _or_ hand-rolled cigarettes?
31.2. Have you _ever_ smoked __beedi__?
31.2.1. How _old_ were you when you first started regular beedi smoking?
31.2.2. Have you ever _stopped_ smoking beedi?
31.2.2.1. How _old_ were you when you last stopped?
31.2.3. On _average_ over the entire time that you smoke(d), about how many beedi per day/per week do (did) you smoke?
31.2.3.1. beedi/day
31.2.3.2. beedi/week
31.3. Have you _ever_ smoked __kreteks__?
31.3.1. How _old_ were you when you first started regular kreteks smoking?
31.3.2. Have you ever _stopped_ smoking kreteks?
31.3.2.1. How _old_ were you when you last stopped?
31.3.3. On _average_ over the entire time that you smoke(d), about how many kreteks per day/per week do (did) you smoke?
31.3.3.1. kreteks/day
31.3.3.2. kreteks/week
31.4. Have you _ever_ smoked __pipes of tobacco__?
31.4.1. How _old_ were you when you first started regular pipe smoking?
31.4.2. Have you ever _stopped_ smoking tobacco pipes?
31.4.2.1. How _old_ were you when you last stopped?
31.4.3. On _average_ over the entire time that you smoke(d), about how many grams per day/per week do (did) you smoke?
31.4.3.1. grams/day
31.4.3.2. grams/week

31.5. Have you _ever_ smoked __cigars, cheroots, or cigarillos__?
31.5.1. How _old_ were you when you first started regular cigar/cheroot/cigarillo smoking?
31.5.2. Have you _stopped_ smoking cigars, cheroots, or cigarillos?
31.5.2.1. How _old_ were you when you last stopped smoking a cigar, cheroot or cigarillo?
31.5.3. On _average_ over the entire time that you smoke(d), about how many cigar/cheroot/cigarillo per day/per week do (did) you smoke?
31.5.3.1. cigars, etc/day
31.5.3.2. cigars, etc/week
31.6. Have you _ever_ smoked a __water pipe__?
31.6.1. How _old_ were you when you first started regular water pipe smoking?
31.6.2. Have you_stopped_ smoking water pipe?
31.6.2.1. How _old_ were you when you last stopped smoking a water pipe?
31.6.3. On _average_ over the entire time that you smoke(d), about how many water pipes per day/per week do (did) you smoke?
31.6.3.1. water pipe/day
31.6.3.2. water pipe/week
31.7. Have you _ever_ smoked __cannabis__?
31.7.1. How _old_ were you when you first started regular cannabis smoking?
31.7.2. Have you _stopped_ smoking cannabis?
31.7.2.1. How _old_ were you when you first started cannabis smoking?
31.7.3. On _average_ over the entire time that you smoke(d), about how many joints/splifs/pipes of cannabis per day/per week do (did) you smoke?
31.7.3.1. joints/day
31.7.3.2. joints/week

31.8. Have you _ever_ vaped/smoked __e-cigarettes__?
31.8.1. How _old_ were you when you first started regular vaping/e-cigarette smoking?
31.8.2. Have you ever _stopped_ vaping/smoking e-cigarettes?
31.8.2.1. How _old_ were you when you last stopped?
31.8.3. On _average_ over the entire time that you vaped/smoke(d), about how many e-cigarettes cartridges per day/per week do (did) you use?
31.8.3.1. e-cigarette cartridges/day
31.8.3.2. e-cigarette cartridges/week
31.8.4. Have you ever vaped/smoked e-cigarettes with aroma(s)?
31.9. Have you ever smoked or inhaled any _other_ substance? (e.g. local, recreational smoked substances)
31.9.1. specify type:
31.9.2. specify unit. e.g. pipes, joints
31.9.3. How_old_ were you when you first started regularly smoking this?
31.9.4. On _average_ over the entire time that you smoke(d), about how many units per day/per week do (did) you smoke?
31.9.4.1. units/day
31.9.4.2. units/week
32. Are you _currently_ smoking anything?
33. How soon after you wake up do you smoke your first cigarette/e-cigarette/beedi/kretek/pipe of tobacco/cigar, cheroot, or cigarillo/water pipe?
34. Do you find it difficult to refrain from smoking in places where it is forbidden (e.g., church, library, cinema, restaurant)?
35. Which cigarette/e-cigarette/beedi/kretek/pipe of tobacco/cigar, cheroot, or cigarillo/water pipe would you be the most unwilling to give up?
36. Do you smoke more frequently during the first hours after waking than during the rest of the day?
37. Do you smoke if you are so ill that you are in bed most of the day?
38. In the last year, how many times have you quit smoking for at least 24 hours?
38.1. Are you seriously thinking of quitting smoking?
38.2. Has a doctor or other health care provider ever _advised_ you to quit smoking?
38.3. Have you received medical advice to stop smoking within the past _12 months_?
38.4. Have you used any medication (prescription or non-prescription), including a nicotine patch, to help you stop smoking?
38.4.1. What kind of _medication_ did you take to help you _stop_ smoking?

38.4.2. Have you used or done _anything else_ to help you stop smoking?
38.4.2.1. What did you do?
39. Has anyone living in your home (besides yourself) smoked a cigarette, pipe or cigar in your home during the past _two weeks_?
39.1. Not counting yourself, how many people in your _household_ smoke regularly?
39.2. Do people smoke regularly in the room where you _work_?
39.3. How many _hours_ per day, are you exposed to other people's tobacco smoke in the following locations?
39.3.1. In the home?
39.3.2. In the workplace?
39.3.3. In bars, restaurants, cinemas or similar social settings?
39.3.4. Elsewhere?
Intro: The following questions refer _only_ to cigarettes and tobacco
39.4. Based on what you know or believe, does smoking tobacco _cause_ serious illness?
39.5. Based on what you know _or_ believe, does smoking tobacco cause the following...
39.5.1. Stroke (blood clots in the brain that may cause paralysis)
39.5.2. Heart attack
39.5.3. Lung cancer
39.5.4. Chronic bronchitis
39.5.5. Emphysema/COPD
40. Have you ever worked for a year or more in a dusty job?
40.1. For how many years have you worked in dusty jobs?
41. Has a doctor or other health care provider ever told you that you had:
41.1. Heart disease
41.1.1. Heart failiure
41.2. Hypertension
41.3. Diabetes
41.4. Lung cancer
41.5. Stroke
**Tuberculosis**
42. Have you _ever_ been diagnosed with __tuberculosis__?
42.1. How many times have you been treated for tuberculosis?
Please answer these questions for the __most recent__ episode.
42.2. When were you last diagnosed as having tuberculosis? (year)
42.3. What part of the body did the tuberculosis affect?
42.4. Were the doctors/clinic _sure_ that you had tuberculosis?
42.4.1. Which tests showed that you had tuberculosis?
42.5. Did you ever stay in hospital for treatment of tuberculosis?

42.5.1. How long for were you in the hospital (sleeping in the hospital)?
42.6. Where did you get your pills or injections for tuberculosis (which clinic)?
42.7. How long _(in months)_ did you take treatment for?
42.8. Did you finish the treatment?
42.8.1. Why did you _not_ complete the treatment?
42.8.2. Did you feel partly or completely well again (better) after ending treatment?
42.8.3. Did the clinic doctor say you were cured?
42.8.4. Did you _stop_ attending the clinic before the treatment was meant to stop?
**Other**
43. Have you ever had an operation on your chest in which a part of your lung was _removed_?
44. In the past 12 months did you get a flu shot?
45. Has a doctor or other health care _professional_ told your father, mother, sister or brother that they had a diagnosis of emphysema, chronic bronchitis or COPD?
**Quality of Life**
The following questions ask for your views about your health—how you feel and how well you are able to do your usual activities. There are no right or wrong answers; please choose the answer that best fits your life right now.
46. In general, would you say your health is: _(Check one)_
47. Does _your health __now__ limit_ you in these activities? If so, how much?
47.1. _Moderate activities_, such as moving a table pushing a vacuum cleaner, bowling or playing golf.
47.2. Climbing _several_ flights of stairs.
48. During _the past 4 weeks_, have you had any of the following problems with your work or other regular daily activities _as a result of your physical health_?
48.1. _Accomplished less_ than you would like.
48.2. Were limited in the _kind_ of work or other activities .
49. During the _past 4 weeks_ have you had any of the following problems with your work or other regular daily activities _as a result of any emotional problems_ (such as feeling depressed or anxious)?
49.1. _Accomplished less_ than you would like.
49.2. Didn't do work or other activities as _carefully_ as usual.
50. During the _past 4 weeks_, how much did pain interfere with your normal work (including both work outside the home and housework)?
These questions are about how you feel and how things have been with you during the past 4 weeks. For each question, please give the one answer that comes closest to the way you have been feeling.
51. How much of the time during the _past 4 weeks_…
51.1. Have you felt calm and peaceful?
51.2. Did you have a lot of energy?

51.3. Have you felt downhearted and blue?
51.4. Have you felt tired?
52. During the _past 4 weeks_, how much of the time has your _physical health or emotional problems_ interfered with your social activities (like visiting friends, relatives, etc.)?
52.1. Compared to one year ago, how would you rate your _physical_ health in general now?
52.2. Compared to one year ago, how would you rate your _emotional_ problems (such as feeling anxious, depressed or irritable) now?
**Work**
The next questions ask about work and about times when you may have missed work due to _health problems_.
53. At any time in the past 12 months, did you work for income?
Paid Employment
53.1. During how many of the past 12 months did you work for income?
53.2. During the months that you worked, how many _days per week_ did you work for income?
53.3. What is the usual number of _hours per day_ you work for income?
53.4. During the past 12 months, did health problems ever stopped you from working for income?
53.4.1. During the past 12 months, how many _total days_ were you unable to work for income due to your health problems?
53.4.2. During the past 12 months, how many _total days_ were you unable to work for income specifically due to breathing problems?
53.5. During the past 12 months, did you not work for income mainly due to _breathing_ problems?
53.6. During the past 12 months, did you not work for income because you were a full-time homemaker or caregiver?
53.7. During the past 12 months, did health problems prevent you from participating in one or more non-work related activities?
53.7.1. During the past 12 months, how many total days did you not participate in non-work related activities due to your health problems?
53.7.2. During the past 12 months, how many _total days_ did you not participate in non-work related activities specifically due to breathing problems?
53.7.3. During the past 12 months, did health problems stop you from performing your usual homemaking/caregiving tasks?
53.7.3.1. During the past 12 months, how many total days were you unable to perform your homemaking/caregiving tasks due to your health problems?
53.7.3.2. During the past 12 months, how many total days were you unable to perform your homemaking/caregiving tasks specifically due to breathing problems?
**Physical activity**
Next, I am going to ask you about the time you spend doing different types of physical activity in a typical week. Please answer these questions even if you do not consider yourself to be a physically active person.

Think about all the _vigorous_ activities that you did in the last 7 days. Vigorous physical activities refer to activities that take hard physical effort and make you breathe much harder than normal. Think only about those physical activities that you did for at least 10 minutes at a time.
54. During the _last 7 days_, on how many days did you do _vigorous_ physical activities like heavy lifting, digging, aerobics, or fast bicycling?
54.1. How much time (hours) did you usually spend doing vigorous physical activities on one of those days?
Think about all the _moderate_ activities that you did in the last 7 days. Moderate activities refer to activities that take moderate physical effort and make you breathe somewhat harder than normal. Think only about those physical activities that you did for at least 10 minutes at a time.
55. During the _last 7 days_, on how many days did you do _moderate_ physical activities like carrying light loads, bicycling at a regular pace, or doubles tennis? Do not include walking.
55.1. How much time (hours) did you usually spend doing moderate physical activities on one of those days?
Think about the time you spent _walking_ in the last 7 days. This includes at work and at home, walking to travel from place to place, and any other walking that you might do solely for recreation, sport, exercise, or leisure.
56. During the _last 7 days_, on how many days did you _walk_ for at least 10 minutes at a time?
56.1. How much time (hours) did you usually spend walking on one of those days?
The last question is about the time you spent _sitting_ on weekdays during the last 7 days. Include time spent at work, at home, while doing course work and during leisure time. This may include time spent sitting at a desk, visiting friends, reading, or sitting or lying down to watch television.
57. During the _last 7 days_, how much time (hours) did you spend _sitting_ on a week day?

1.3 Appendix C: Verbal autopsy questionnaire

**Identification**
Site
Fieldworker number
Participant number
**Status of participant**
1. Can the participant be contacted?
1.1. Why can't the participant be contacted?
1.2. When was this person last heard from?
**Cause of death**
2. What is the relation between the deceased and the informant?
3. Is there a death certificate?
**Death Certificate**
3.1. What is the date of death on the certificate?
3.2. Is the ICD code available
ICD Codes
3.2.1.1. Record the immediate cause of death from the certificate.
3.2.1.2. Record the first underlying cause of death from the certificate.
3.2.1.3. Record the second underlying cause of death from the certificate.
3.2.1.4. Record the first contributing cause of death from the certificate.
3.2.1.5. Record the second contributing cause(s) of death from the certificate.
Uncoded causes of death
3.2.2.1. Record the immediate cause of death from the certificate.
3.2.2.2. Record the first underlying cause of death from the certificate.
3.2.2.3. Record the second underlying cause of death from the certificate.
3.2.2.4. Record the third underlying cause of death from the certificate.
3.2.2.5. Record the contributing cause(s) of death from the certificate.
**Verbal Autopsy**
****SECTION 1: INJURIES AND ACCIDENT****
4. Did [*participant's name*] suffer from an injury or accident that led to his/her death?
4.1. What kind of injury or accident did [*participant's name*] suffer from?
4.1.1. Specify other:
4.2. Was the injury or accident self-inflicted?

4.3. Was the injury or accident intentionally inflicted by someone else?
**SECTION 2: HISTORY OF CHRONIC CONDITIONS OF THE DECEASED**
5. Was [*participant's name*] ever told by a health professional that he or she ever suffered from one of the following?
5.1. Asthma
5.2. Cancer
5.3. COPD (Chronic Obstructive Pulmonary Disease)
5.4. Diabetes
5.5. Epilepsy
5.6. Heart Disease
5.7. Tuberculosis
5.8. Stroke
5.9. AIDS
**SECTION 3: SYMPTOM CHECKLIST**
These questions pertain mainly to the 12 months prior to the death of [*participant's name*]
6.1. Did [*participant's name*] have a fever?
6.1.1. How severe was the fever?
6.1.2. What was the pattern of the fever?
6.2. Did [*participant's name*] have a rash?
6.2.1. Where was the rash located?
6.3. Did [*participant's name*] have sores?
6.3.1. Did the sores have clear fluid or pus?
6.4. Did [*participant's name*] have an ulcer (pit) on the foot?
6.4.1. Did the ulcer ooze pus?
6.4.2. For how many days did the ulcer ooze pus?
6.5. Did [*participant's name*] experience “pins and needles” in their feet?
6.6. Did [*participant's name*] have blue lips?
6.7. Had [*participant's name*] lost weight in the three months prior to death?
6.7.1. How substantial was the loss of weight?
6.8. Did [*participant's name*] look pale?
6.9. Did [*participant's name*] have yellow discoloration of the eyes?
6.9.1. For how long did [*participant's name*] have the yellow discoloration?
6.9.1.1. Days:
6.9.1.2. Months:
6.10. Did [*participant's name*] have ankle swelling?
6.10.1. For how long did [*participant's name*] have ankle swelling?
6.10.1.1. Days:
6.10.1.2. Months:

6.11. Did [*participant's name*] have puffiness of the face?
6.11.1. For how long did [*participant's name*] have puffiness of the face?
6.11.1.1. Days:
6.11.1.2. Months:
6.12. Did [*participant's name*] have general puffiness all over his/her body?
6.12.1. For how long did [*participant's name*] have puffiness all over his/her body?
6.12.1.1. Days:
6.12.1.2. Months:
6.13.1. Did [*participant's name*] have a lump in the neck?
6.13.2. Did [*participant's name*] have a lump in the armpit?
6.13.3. Did [*participant's name*] have a lump in the groin?
6.14. Did [*participant's name*] have a cough?
6.14.1. For how long did [*participant's name*] have a cough?
6.14.1.1. Days:
6.14.1.2. Months:
6.14.2. Did the cough produce sputum?
6.15. Did [*participant's name*] cough blood?
6.16. Did [*participant's name*] have difficulty breathing?
6.16.1. For how long did [*participant's name*] have difficulty breathing?
6.16.1.1. Days:
6.16.1.2. Months:
6.16.2. Was the difficulty continuous or on and off?
6.16.3. In what position did the difficulty get worse?
6.17. Did [*participant's name*] have fast breathing?
6.17.1. For how long did [*participant's name*] have fast breathing?
6.17.1.1. Days:
6.17.1.2. Months:
6.18. Did [*participant's name*] wheeze?
6.19. Did [*participant's name*] experience pain in the chest in the month preceding death?
6.19.1. How long did the pain last?
6.19.2. Was the pain during physical activity?
6.19.3. Where was the pain located?
6.20. Did [*participant's name*] have more frequent loose or liquid stools than usual?
6.20.1. Was there blood in the stool?
6.20.2. Was there blood in the stool up until death?
6.21. Did [*participant's name*] stop urinating?

6.22. Did [*participant's name*] vomit in the week preceding the death?
6.22.1. Was there blood in the vomit?
6.22.2. Was the vomit black?
6.23. Did [*participant's name*] have difficulty swallowing?
6.23.1. For how long before death did [*participant's name*] have difficulty swallowing?
6.23.1.1. Days:
6.23.1.2. Months:
6.23.2. Was the difficulty with swallowing with solids, liquids, or both?
6.23.3. Did [*participant's name*] have pain upon swallowing?
6.24. Did [*participant's name*] have belly pain?
6.24.1. For how long before death did [*participant's name*] have belly pain?
6.24.1.1. Hours:
6.24.1.2. Days:
6.24.1.3. Months:
6.24.2. Was the pain in the upper or lower belly?
6.25.Did [*participant's name*] have a more than usual protruding belly?
6.25.1. For how long before death did [*participant's name*] have a protruding belly?
6.25.1.1. Days:
6.25.1.2. Months:
6.25.2. How rapidly did [*participant's name*] develop the protruding belly?
6.26. Did [*participant's name*] have any mass in the belly?
6.26.1. For how long before death did [*participant's name*] have a mass in the belly?
6.26.1.1. Days:
6.26.1.2. Months:
6.27. Did [*participant's name*] have a stiff neck?
6.27.1. For how long before death did [*participant's name*] have stiff neck?
6.27.1.1. Days:
6.27.1.2. Months:
6.28. Did [*participant's name*] experience a period of loss of consciousness?
6.28.1. Did the period of loss of consciousness start suddenly or slowly?
6.28.2. Did it continue until death?
6.29. Did [*participant's name*] have convulsions?
6.29.1. For how long before death did the convulsions last?
6.29.1.1. Minutes:
6.29.1.2. Hours:
6.29.2. Did the person become unconscious immediately after the convulsions?

6.30. Was [*participant's name*] in any way paralyzed?
6.30.1. Which were the limbs or body parts paralyzed?
6.31. Was the participant a woman?
****SECTION 4: QUESTIONS FOR WOMEN****
**Questions for women only**
7.1. Did [*participant's name*] have any swelling or lump in the breast?
7.2. Did [*participant's name*] have any ulcers (pits) in the breast?
7.3. Had [*participant's name*]'s periods stopped naturally because of menopause?
7.4. Did [*participant's name*] have vaginal bleeding after cessation of menstruation? (post-menopausal)
7.5. Was she pregnant at the time of death or in the 6 weeks prior to her death?
7.5.1. For how many months was she pregnant?
7.6. Did she die during labour or delivery?<br>("Labour" is the period of time by which contractions are less than 10 minutes apart.)
**Tobacco**
8. Did [*participant's name*] use tobacco?
**Healthcare and Health Records**
9. Was care sought outside the home while the deceased had this illness?
Healthcare
9.1. Where or from whom did [*participant's name*] seek care?<br>(CHECK ALL THAT APPLY)
9.2. Record the name and address of the government hospital where the care was sought:
9.3. Did a health care worker tell you the cause of death?
9.3.1. What did the health care worker say?

1.4 Appendix D: Chichewa translation of the Verbal autopsy questionnaire.

**Zodziwitsa**
Malo
Namabala ya wogwira nchito ku dera
Namabala ya wotenga nawo mbali
**Zokhudzana ndi wotenga nawo mbali**
1.. Kodi wotenga nawo mbali tingalumikizane naye?
1.1. Chifukwa chani wotenga nawo mbali sitingalumikizane naye ?
1.2. Munthu womaliza munalankhula naye liti?
**Chimene chinabweretsa imfa**
2. Kodi pali chibale chotani pakati pa munthu amene akupereka uthenga ndi malemu?
3. Pali chikalata chotsimikiza za imfa cha kuchipatala ?
**Chikalata chotsimikiza imfa cha ku chipatala.**
3.1.Pali deti lanji pa chiphatso
3.2 kodi ICD code ilipo
ICD CODES
3.2.1.1. Lembani chimene chinabweretsa imfa kumapeto kuchokera pa chikalata cha kuchipatala
3.2.1.2.Lembani chinthu choyamba choganiziridwa chimene chinalebedwa kuti chinabweretsa imfa kuchokera pa chikalata cha kuchipatala
3.2.1.3. Lembani chinthu chachiwiri choganizirirdwa chimene chinalembedwa kuti chinabweretsa imfa kuchokera pa chikalata
3.2.1.4. Lembani chinthu choyamba chenicheni chimene chinabweretsa imfa kuchokera cha chikalata cha ku chipatala
3.2.1.5. Lembani chinthi chachiwiri
Zosalembedwa zobweretsa imfa
3.2.2.1 Lembani chimene chinabweretsa imfa kumapeto kuchokera pa chikalata cha kuchipatala
3.2.1.2.Lembani chinthu choyamba choganiziridwa chimene chinalebedwa kuti chinabweretsa imfa kuchokera pa chikalata cha kuchipatala
3.2.1.3. Lembani chinthu chachiwiri choganizirirdwa chimene chinalembedwa kuti chinabweretsa imfa kuchokera pa chikalata
3.2.1.4. Lembani chinthu choyamba chenicheni chimene chinabweretsa imfa kuchokera cha chikalata cha ku chipatala
3.2.1.5. Lembani chinthi chachiwiri
**Kufufuza za imfa polankhulana**
****Gawo 1: ZOVULALA NDI NGOZI**
4. Kodi [*dzina la wotenga nawo mbali*] anavulala kapena anachita ngozi imene inabweretsa imfa?

4.1. Kodi anavulala mwa mtundu wanji kapena anachita ngozi ya mtundu wanji [*dzina la wotenga nawo mbali*]?
4.1.1. Fotokozani zina :
4.2. Kodi kuvulalako anazipanga yekha kapena ngoziyo anayambitsa yekha?
4.3 Kodi kuvulala kunapangidwa ndi munthu wina kapena ngoziyo inapangidwa ndi munthu wina ?
**GAWO 2: MBIRI YA MATENDA A M'GONAGONA YA MALEMU**
5. Kodi [*dzina la wotenga nawo mbali nawo mbali**] anauzudwapo ndi a chipatala kuti iye amadwala chimodzi mwa zotsatirazi?
5.1 Mphumu
5.2 Kkhansa
5.3 Matenda a mgonagona a m'mapapo kapena mtima (COPD)
5.4 Matenda a shuga
5.5 Khunyu
5.6 Matenda a mtima
5.7Chifuwa chachikulu
5.8 Kufooka ziwalo
5.9 Edzi
**GAWO 3: M'NDANDANDA WA ZIZINDIKIRO
Mafunso amenewa akhudza m'mene munthu analili miyezi 12 asanamwalire [*dzina la wotenga nawo mbali*]
6.1 Kodi [*dzina la wotenga nawo mbali *] anatentha thupi ?
6.1.1 . Thupi linatentha kwambiri bwanj?
6.1.2Kodi kutentha thupi kumakhala bwanji?
6.2 Kodi [*wotenga nawo mbali*] anatuluka ziwengo?
6.2.1 Ziwengo zinali pati?
6.3 Kodi [*dzina la wotenga nawo mbali*] anali ndi zilonda?
6.3.1 Zilonda zianali ndi madzi kapena mafinya?
6.4. Kodi [*dzina ala wotemga nawo mbali*] anali ndi bala (lolowa) pa phanzi?
6.4.1 Bala limakha mafinya?
6.4.2 Bala linakha madzi mafinya angati?
6.5 Kodi [*dzina la wotenga nawo mbali*] amamva kubaya baya ngati "masingano" ku mapanzi awo?
6.6 Kodi [*dzina la wotenga nawo mbali*[anali ndi milomo ya blue.
6.7 [*dzina la wotenga nawo mbali*] anawonda m'miyezi itatu yomaliza asanamwalire?
6.7.1. Kodi anawonda muyezo wotani?
6.8 [*dzina la wotenga nawo mbali*] amaoneka kuti akusowa magazi?
6.9. [*dzina la wotenga nawo mbali*] anali ndi maso achikasu?
6.9.1 Kodi [*dzina la wotenga nawo mbali*] anakhala ndi maso a chikasu kwa nthawi yaitali bwanji?
6.9.1.1 Masiku:
6.9.1.2. Miyezi
6.10. [*dzina la wotenga nawo mbali *] amatupa mu kamfula?
6.10 [*dzina la wotenga nawo mbali*] watupa mu kamfula kwa nthawi yaitali bwanji?
6.10.1.1.Masiku

6.9.1.2 Miyezi
6.11. Kodi [*dzina la wotenga nawo mbali*]amatupa nkhope?
6.11.1 [*dzina la wotenga nawo mbali*] anatupa nkhope kwa nthawi yaitali bwanji?
6.11.1.1 Masiku
6.11.1.2 Miyezi
6.12 [*dzina la wotenga nawo mbali*] amatupa thupi lake lonse?
6.12.1 [*dzina la wotenga nawo mbali*] anatupa thupi lake lonse kwa nthawi yaitali bwanji ?
6.12.1.1 Masiku
6.12.1.2. Miyezi
6.13.1. Kodi [*dzina la wotenga nawo mbali*] anali ndi chotupa pakhosi?
6.13.2. Kodi [*dzina la wotenga nawo mbali*] anali ndi chotupa pa mkono?
6.13.3. kodi [*dzina la wotenga nawo mbali*] anali ndi chotupa pakati pa mwendo ndi mimba?
6.14. [*dzina la wotenga nawo mbali*] anali ndi chifuwa ?
6.14.1. [*dzina la wotenga nawo mbali*anadwla nthawi yaitali bwanji?
6.14.1.1. Masiku :
6.14.1.2. Miyezi
6.14.2. Kodi chifuwa chimatulutsa makhololo?
6.15 Kodi [*dzina la wotenga nawo mbali*] amakhosomola magazi
6.16 Kodi [*dzina la wotenga nawo mbali*]anali ndi mavuto popuma?
6.16.1 [*dzina la wotenga nawo mbali*] anali ndi mavuto pouma kwa nthawi yaitali bwanji?
6.16.1.1 Masiku :
6.16.1.2. Miyezi
6.16.2. Kodi kuvuka popuma kunali kongopitirira kapena mumabwera panthawi?
6.16.3. Kodi amavutika kwambiri akhala bwanji?
Kodi [dzina la wotenga nawo mbali*] amapuma mothamanga ?
6.17.1[*dzina la wotenga nawo mbali*] amapuma mothamanga kwa nthawi yaitali bwanji?
6.17.1.1.Masiku
6.17.1.2. Miyezi
6.18. Kodi [*dziana la wotenga nawo mbali*] amalira kukhosi popuma?
6.19 Kodi [*dzina la wotenga nawo mbali*] amamva kupweteka m'chifuwa mu mwezi umene anamwalira?
6.19.1 Kodi kupweteka kumeneku kunatenga nthawi yaitali bwanji?
6.19.2. Kodi kupweteka kumabwera pogwira ntchito ?
6.19.3. Kodi kupwetekakuku kunali patipo?
6.20 Kodi [*dziana la wotenga nawo mbali*0 amachita chimbudzi cha madzi kawirikawiri kuposera masiku onse?
6.20.1 Kodi mu chimbudzi mumakhala magazi
6.20.2 Kodi mu chimbudzi mumakhala magazi mpaka nthawi yomwalira?
6.21 Kodi [*dzina la wotenga nawo mbali*] anasiya kukodza?
6.22 Kodi[dzina la wotenga nawo mbali*] amasanza mu sabata imene anamwalira
6.22.2 Kodi munali magazi mu masanzi?
6.22.2. Kodi masanzi anali anali akuda?

6.23 Kodi[*dzina la wotenga nawo mbali*] anali ndi mavuto pomeza?
6.23.1.[*dzian la wotenga nawo mbali*] anali ndi mavuto pomeza kwa nthawi yaitali bwanji asanamwalire ?
6.23.1.1 Masiku
6.23.1.2.
6.23.2. Kodi amavutika kumeza zolimba, zamadzi kapena zonse?
6.23.3 [*dzina la wotenga nawo mbali*] amava kupweteka pomeza?
6.24. [*dzina la wotenga nawo mbali*] amava kupweteka m'mimba?
6.24.1. [*dzina la wotenga nawo mbali*] anamva kupweteka m'mmba kwa nthawi yaitali bwanji?
6.24.1.1. maola
6.24.1.2. Masiku
6.24.1.3. miyezi
6.24.2 Kodi ululu unali m'mwamba kapena m'munsi mwa mimba?
6.25 [*dzina la wotenga nawo mbali*] anakula mimba kuposera mmene inalili?
6.25.1Kodi mimba inakhala yotupa kwa nthawi yaitali bwanji?
6.25.1.1 Masiku
6.25.1.2 Miyezi
6.25.2 . Kodi mimba ya [*dzina la wotenga nawo mbali *] imakula mwachangu bwanji?
6.26 Kodi m'mimba mwa [*dzina la wotenga nawo mbali*] munali madzi?
6.26.1. [*dzina la wotenga nawo mbali*] anakhala ndi madzi m'mimba kwa miyezi ingati ?
6.26.1.1 Masiku
6.26.1.2. Miyezi
6.27. Kodi [*dzina la wotenga nawo mbali*] amauma khosi?
6.27.1. Kodi [*dzina la wotenga nawo mbali anakhala ndi khosi louma kwa nthawi yaitali bwanji ?
6.27.1.1 Masiku [*Dzina l
6.27.1.2 Miyezi
6.28. Kodi [*dzina la wotenga nawo mbali*] ankhalapo chikomokere
6.28.1 Kodi nthawi imene anakhala chikomokere chinangochitika mwadzidzidzi kapena zinayamba pang'onopang'ono/
6.28.2. Kodi zinapiritiria mpaka anamwalira ?
6.29. Kodi [*dzina la wotenga nawo mbali*] amakomoka komoka ?
6.29.1 Kodi amakomokakomoka kwa nthawi yaitali bwanji asanamwalire?
6.29.1.1 Mpindi
6.29.1.2. Maola
6.29.2.Kodi muthuyo anakhala chikomokere zitangochitika zokomokakomoka?
6.30 Kodi [*dzina la wotenga nawo mbali*] anafooka ziwalo?
6.30.1. Kodi ndi ziwalo ziti kaoena mbali iti imene inafooka?
6.31. Kodi wotenga nawo mbali anali wamkazi kapena wammuna ?
****GAWO 4: MAFUNSO A AKAZI**
**Mafunso okhudza amai basi**
7.1 Kodi [dzina la wotenga nawo mbali*] anali ndi zotupa kapena mbulu mu bere?
7.2 [*dzina la wotenga nawo mbali*] anali ndi mabala (olowa) m'mabere ?

7.3 Kodi [*dzina la wotenga nawo mbali*] anasiya kusamba chifukwa nthawi inakwana ?
7.4[*dzina la wotenga nawo mbali*] amasamba atasiya kale kusamba ?
7.5 .Kodi anali ndi mumba pamene amawalira kapena miyezi isanu ndi umodzi asanamwalire ?
7.6 Kodi anamwalira panthawi imene anali pa matenda kapena pobereka?
('pa matenda" ndi nthawi imene kupweteka kumakhala kotalikirana mpindi khumi)
**FODYA**
8.Kodi [*dzina la wotenga nawo mbali *] amasuta fodya?
**Chisamalira cha ku chipatala ndi zolemba zokhudzana ndi umoyo**
9. Kodi chisamaliro chimachokera kunja pamene malemu amadwala?
**Chisamaliro cha kuchiapatala**
9.1 [*dzina la wotenga nawo mbali*] Amapeza chisamaliro kuti? (ONANI ZIMENE ZIKUGWIRIZANA)
9.2 Lembani dzina ndi keyala ya chipatala cha boma kumene anakalandira chithandizo:
9.3 Kodi ogwira ntchito za umoyo anakuuzani chimene chinabweretsa imfa ?
9.3.1. Kodi wogwira ntchito za umoyo anati chani?

1.5 Appendix E: Chichewa translation of the Core questionnaire.

NDONDOMEKO YA MAFUNSO A BOLD CORE

Demographics

1. Kodi wolowanawo mukafukufukuyi ndi mwamuna kapena mkazi? Mwamuna ☐

    mkazi ☐

2. Kodi ndinu mtundu wanji wa anthu? __________________ ___ ___

3. Kodi munabadwa liti? ____ ____/____ ____/____ ____ ____ ____
    d d m m y y y y

4. Kodi sukulu munalekezera pati? (Zaka) ___ ___

5. Kodi maphunziro anu mwafika nawo pati Pakali ano? Pulayimale sukulu ☐
    Sekondale sukulu ☐
    Ma koleji ena ☐
    Sukulu ya ukachenjede/umisili ☐
    palibe ☐
    sizikudziwika ☐

6. Kodi abambo anu maphunzilo awo adalekezera pati? Pulayimale sukulu ☐

    Sekondale sukulu ☐
    Makoleji ena (Trade/Professional/Community) ☐
    Koleji ya ukachenjede/umisili ☐
    palibe ☐
    sizikudziwika ☐

6.1 Kodi amai anu maphunziro awo adelekezera pati? Pulayimale sukulu ☐

    Sekondale sukulu ☐
    Makoleji ena ☐
    Kolei yaukachenjede/yaumisili ☐
    palibe ☐
    sizikudziwika ☐

6.2. Chonde tandiuzeni ngati nyumbayi kapena wina aliyense nyumbamu ali/anagula zinthu izi werengani chinthu chinachilichonse:

	EYA	AYI	SIZIKUDZIWIKA
a. magetsi?	❑	❑	❑
b. chimbudzi chogejemula?...	❑	❑	❑
c. lamya wa pansi	❑	❑	❑
d. lamya ya m'manja?	❑	❑	❑
e. kanema?	❑	❑	❑
f. wailesi?	❑	❑	❑
g. friji?	❑	❑	❑
h. galimoto?	❑	❑	❑
i. Thutumula ya moto?	❑	❑	❑
j. Makina wochapila zovala?	❑	❑	❑
k. nyumba yawo yogula okha.............................	❑	❑	❑
l. bafa ya m'nyumba kapena shawa............................	❑	❑	❑
m. mpope wa madzi wa nyumba...	❑	❑	❑
n. mpope wapanja wawo wawo........................	❑	❑	❑

q. Mu chaka chapitachi inuyo kapena wina aliyense wa nyumba mwanu, alipo amene anakhapo ndi njala chifukwa chosowa ndalama?

☐ masiku ambiri
☐ milungu ya mbiri
☐ miyezi ya mbiri
☐ nthawi zina pa chaka
☐ nthawi ndi nthawi
☐ ndikale lonse...............................

6.3 Pa nthawi yomwe munali ndi zaka zisanu alipo amene mumakhala naye nyumba mwanu limodzi anali ndi/anagula zinthu izi;

Werengani chinachilichonse	EYA	AYI	SIZIKUDZIWIKA
a. magetsi?	❑	❑	❑
b. chimbudzi chogejemula?	❑	❑	❑
c. l amya yapansi?	❑	❑	❑
d. kanema wa zinthunzi?	❑	❑	❑
e. wailesi?	❑	❑	❑
f. firiji?	❑	❑	❑
g. galimoto?	❑	❑	❑
h. Moped/scooter/njinga ya moto?	❑	❑	❑
i.makina wochapila zovala?	❑	❑	❑
j. kugula nyumba yawo yawo..........................	❑	❑	❑
k. bafa ya m'nyumba kapena shawa.......................	❑	❑	❑
l. mpope wa m'nyumba...	❑	❑	❑
m. mpope wa panja wawowawo...................	❑	❑	❑

p. Kukhala ndi njala chifukwa chosowa ndalama?
masiku ambiri
milungu ya mbiri
miyezi ya mbiri
nthawi zina pa chaka
mwa panthawi
ndi kale lonse...............................

6.4. Kodi mukhala anthu angati m'nyumba mwanu(kuphatikizirapo inuyo) __ __

6.5. Kodi nyumba yanu ili ndi zipinda zingati? (osawelengera kitchini kapena bafa) __ __

Zizindikilo ndi matenda a kapumidwe

Mafunso awa ndi okhudzana mchifuwa chanu. Chonde yankhani kuti eya kapena ayi ngati kuli kotheka. Ngati mukaikila kuti yankho lanu ndi eya kapena ayi chonde lembani kuti ayi.

kukhosmola

7. Kodi nthawi zambiri mumakhosmola pamene mulibe chifuwa? Eya
Ayi
[ngati eya , pitilizani ndi funso nambala 7A; ngati ayi pitani ku funso nambala 8]

7A. Kodi pali <u>miyezi</u> imene mumakhosomola <u>masiku ambiri</u>? Eya
Ayi
[ngati ayankha eya, funsani mafunso <u>anse</u> awiri funso nambala 7B ndi 7C; ngati ayankha ayi pitaniku funso nambala 8]

7B. Kodi mumakhosomola <u>masiku ambiri</u> okwanila miyezi itatu pa chaka chinachilichonse? Eya
Ayi

7C. Kodi ndi kwa zaka zingati zimene mwakhala ndi chifuwa chimenechi? Kwa zaka zochepera ziwiri
pakati pa zaka ziwiri ndi zisau
pyolora zaka zisanu

<u>makhololo</u>

8. Kodi nthawi zambiri mumatulitsa makhololo anu kuchokera mchifuwa, kapena nthawi zambiri mumakhala ndi makhololo amene mumavutika kuwatulutsa Eya ☐

ngati musakudwala chifuwa? Ayi ☐

[ngati ayankha eya pitilizani ndifunso nambala 8A; ngati ayankha ayi, pitani ku funso nambala 9]

8A. Kodi pali miyezi ina imene mumatulutsa makhololo masiku ambiri Eya ☐
Ayi ☐

[ngati eya funsani mafunso onse awiri, funso nambala 8B ndi 8C; ngati ayi pitani kufunso nambala 9]

8B. Kodi mumatulutsa makhololo amenewa kwa masiku ambiri mwina kwa miyezi itatu pa chaka? Eya ☐
Ayi ☐

8C. Kodi ndi kwa zaka zingati zomwe mwakhala ndi makhololo? Kosachepera zaka ziwiri ☐

pakati ma zaka ziwiri ndi zisanu ☐
kupyolera zaka zisanu ☐

Kusokosera mpweya m'chifuwa

9. kodi munanvapo kusokosera mpweya (wheezing or whistling) m'chifuwa mwanu nthawi ina
iliyonse kwa miyezi khumi ndi iwiri yapitayi ? Eya ☐
Ayi ☐

[ngati ayankha eya, funsani mafunso onse nambala 9A ndi nambala 9B; ngati ayankha ayi, pitani ku funso nambala 10]

9A. Kwa miyezi khumi ndi iwiri yapitayi, munanvapo kusokosera mpweya (wheezing or whistling) m'chifuwa mwanu pokhapokha mutadwala chifuwa? Eya ☐

Ayi ☐

9B. Kwa miyezi khumi ndi iwiri yapitayi, munanvapo kusokosera mpweya (wheezing or whistling) m'chifuwa zimene zinakupangitsani kubanika? Eya ☐
Ayi ☐

Kubanika

10. Kodi mumalephera kuyenda chifukwa cha zinthu zina kapena matenda ena kupatula kubanika Eya
Ayi

[Ngati ayankha eya ku funso nambala 10, chonde longosolani bwinobwino za vutoli mzere uli munsiwu ndipo pitani pa funso nambala 12. Ngati yankho lili ayi kapena akukaikila, pitani pa funso nambala 11.]

vuto: ___________________

11. Kodi mumavutika ndi kubanika pamene mukuyenda mofulumira pa malo a fulati kapena mukuyenda mtunda waung'ono? Eya
Ayi
[ngati ayankha eya, funsani funso nambala 11A mpakana 11D; ngati ayankha kuti ayi, pitani pa funso nambala 12]

11A. Kodi mumayenera kuyenda pang'onopang'ono kusiyana ndi anthu a msinkhu wanu pa malo opanda zitunda chifukwa Cho banika? Eya
Ayi
Sizikugwilizaana

11B. Kodi mumapumira kaye mukamyenda pa mulingo wanu pa malo opanda zitunda? Eya
Ayi
Sizikugwilizaana

11C. Kodi mumayenera kupumira mutayenda mtunda wokwanira 100 (kapena kwa mpindi zochepa) kwa malo opanda zitunda? Eya
Ayi
Sizikugwilizaana

11D. Kodi mumabanika mukamatuluka nyumba kapena panthawi imene mukuvala kapena mukuvula chovala? Eya
Ayi
Sizikugwilizaana

12. Kodi adokotala kapena ena a zaumoyo anayambapo atakuwuzanipo kuti muli ndi nthenda ya m'mapapo yomwe imapangitsa kuti mubanike? Eya ☐

Ayi ☐

13. Kodi adokotala kapena ena azaumoyo ankuwuzaniponi kuti muli ndi nthenda ya mphumu, chifuwa chokhudzana ndi mphumu, kapena matenda ena aliwonse okhudzana ndi njira yopumira? Eya ☐

Ayi ☐

[ngati ayankha eya, funsani funso nambala 13A. Ngati ayankha ayi pitani ku funso nambala 14]

13A. Kodi mukanali ndi nthenda ya mphumu, chifuwa chokhudzana ndi mphumu,kapena matenda ena aliwonse okhudzana ndi njira yopumira?

Eya ☐

Ayi ☐

14. Kodi adokotala kapena a zaumoyo anyambapo akuwuzani kuti muli ndi matenda a chifuwa cha m'gona gona Eya ☐

Ayi ☐

[ngati ayankha kuti eya, funsani funso nambala 14A. Ngati aynkha kuti ayi, pitani ku funso nambala 15]

14A. Kodi mukadali nayo nthenda ya chifuwa cha m'gona gona Eya ☐

Ayi ☐

15. Kodi adokotala kapena a zaumoyo anayamba akuwuzaniponi kuti muli ndi nthenda yam'gonagona yokhudzana ndi kutsekeka kwa njira yopumira? Eya ☐

(COPD) Ayi ☐

Management Section

Pano ndikufunsani zokhudzana mankwala amene mukhonza kumwa kuti akuthandizeni ku mapumidwe. Ndikufuna kuti ndidziwe za mankhwala amene mumamwa kawirikawiri komanso mankhwala amene mukhonza kumwa kuti mungochepetsa ululu wa vutolo. Ndikufuna mundiwuze mankhwala ena aliwonse amene mumamwa, njira yake komanso

mumagwritsa ntchito mowirikiza bwanji pa mwezi wina uliwonse.

16. Mu miyezi khumi ndi iwiri yapitayi, kodi mwamwapo mankhwala a vuto la mapumidwe kupahatikizapo mankhwala Oletsa kuti phuno zisatseke? Eya ☐
Ayi ☐

Ngati otenga nawo mbali sakumwa mankhwala ena aliwonse owathandiza mbali ya mapumidwe awo, pitani ku funso nambala 17.

16A. Dzina la Mankhwala (sinalewmbed we)							
16B. Nambala ya mankhwala	___ ___ ___	___ ___ ___	___ ___ ___	___ ___ ___	___ ___ ___	___ ___ ___	___ ___ ___
16C.Kapangid we	mb'ulu wa ❑<br>mankhwala ❑<br>Inhaler ❑<br>Nebulizer ❑<br>A madzi ❑<br>Suppositor ❑<br>Jakisoni ❑<br>Ena	mb'ulu ❑<br>wmankhwa ❑<br>Inhaler ❑<br>Nebulizer ❑<br>A madzi ❑<br>Suppositor ❑<br>Jakisoni ❑<br>Ena	mb'ulu wa ❑<br>mankhwala ❑<br>Inhaler ❑<br>Nebulizer ❑<br>A madzi ❑<br>Suppositor ❑<br>Jakisoni ❑<br>Ena	mb'ulu wa ❑<br>mankhwala ❑<br>Inhaler ❑<br>Nebulizer ❑<br>A madzi ❑<br>Suppositor ❑<br>Jakisoni ❑<br>Ena	mb'ulu wa ❑<br>makhwala ❑<br>Inhaler ❑<br>Nebulizer ❑<br>Amadzi ❑<br>Suppositor ❑<br>Jakisoni ❑<br>Ena	mb'ulu wa ❑<br>mankhawa ❑<br>Inhaler ❑<br>Nebulizer ❑<br>Amadzi ❑<br>Suppositor ❑<br>Jakisoni ❑<br>Ena	mb'ulu wa ❑<br>mankhwala ❑<br>Inhaler ❑<br>Nebulizer ❑<br>Amadzi ❑<br>Suppositor ❑<br>Jakisoni ❑<br>Ena
16D. Kodi mankhwala wo amamwedwa masiku onse kapena nthawiyomwe muli ndi zizindikilo, kapena zonse (*ngati ayankha kuti 'masiku onse' afunseni fnso nambala 16 16 F , ngati ayankha kuti*	❑<br>Masiku ons ❑<br>zizindikilo ❑<br>zonse ❑<br>zina	❑<br>Masiku on ❑<br>zizindikilo ❑<br>zonse ❑<br>zina	❑<br>Masiku or ❑<br>zizindikilo ❑<br>zonse ❑<br>zina	❑<br>Masiku or ❑<br>zizindikilo ❑<br>zonse ❑<br>zina	❑<br>Masiku or ❑<br>zizindikilo ❑<br>zonse ❑<br>zina	❑<br>Masiku or ❑<br>zizindikilo ❑<br>zonse ❑<br>zina	❑<br>Masiku or ❑<br>zizindikilo ❑<br>zonse ❑<br>zina

'zonse' funsani mafunso onse funso nambala 16E ndi 16 F)							
16E. Nthawi yomwe mukumwa mankhwala, kodi mumamwa masiku angati pa mulungu?	__masiku/mulungu	__masiku/mulungu	__masiku/mulungu	__masiku/mulungu	__masiku/mulungu	__masiku/mulungu	__masiku/mulungu
16F. Nthawi yomwe munamwa mankhwalawo, kodi ndi miyezi ingati pa miyezi khumi yapitayi imene mwamwa mankhwalawo?	0-3 4-6 7-9 10-12	0-3 4-6 7-9 10-12	0-3 4-6 7-9 10-12	0-3 4-6 7-9 10-12	0-3 4-6 7-9 10-12	0-3 4-6 7-9 10-12	0-3 4-6 7-9 10-12

17. chonde tandiwuzeni zinthu zomwe mumamwa kapena zomwe mumachita kuti zikuthandizeni kumbaliya vuto lamapumidwe zimene simunandiwuze

Mankhwala kapena zochita	nambala
	____ ____ ____
	____ ____ ____
	____ ____ ____
	____ ____ ____

18. Kodi adokotala kapena azaumoyo anakuyezaniponi mapapo ndi makina oyezera mpweya? (mwachitsanzo spirometer or peakflow meter) Eya ☐

Ayi ☐

[ngati ayankha kuti eya,funsani funso nambala 19A Ngati ayankha kuti ayi, pitani ku funso nambala 19]

18A. Kodi mwagwiritsapo ntchito makina amenewa pa miyezi khumi ndi iwiri yapitayi? Eya ☐
Ayi ☐

19. Kodi munali ndi nthawi imene munavutika kupuma kwambiri mpakana kuti kunali kovuta kuti mugwire ntchito zanu za tsiku ndi tsiku kapena kulephera kupita ku ntchito? Eya ☐
Ayi ☐

[ngati yankho lili eya, funsani funso nambala 19A Ngati anena kuti ayi, pitani kufunso nambala 20]

19A. Kodi vuto ngati limeneli munakhala nalo kangati miyezi khumi ndi iwiri yapitayi? ____ ____ ____ vutolo

[ngati anali ndi vuto limeneli, funsani funso nambala 19B ndi 19C ngati ayi pitani ku funso nambala 20]

19B. Pa mavuto amenewa ndi kangati munawonana ndi adotolo kapena ogwira ntchito m'chipatala kwa miyezi Khumi ndi iwiri yapitayi? _____ _____ vutoli

19C. Pa mavuto amenewa kodi munayamba mwago nekedwapo m'chipatala miyezi khumi ndi iwiri yapitayi?

_____ _____   vutoli

[ngati 19C >0, funsani funso nambala 19C1, else pitani ku funso nambala 20]

19C1. Kodi ndi masiku angati amene munagonekedwa
m'chipatala chifukwa cha vuto la mapumidwe
mu miyezi khumi ndi iwiri yapitayi   ____ ____ ____ masiku

kusuta fodya

20. Tsopano ndikufansani zosuta fodya. Poyambilira ndikufunsani za ndudu, kuphatikiza wopichila nokha, kenaka ndizakufunsani za za fodya wina amene amasutidwa.

20.1. Kodi munayambapo mwasutapo fodya?   Eya ☐
Ayi ☐

("eya" amatanthauza ma paketi a ndudu opyolera makumi awiri mu moyo wawo kapena ndudu yopyolera imodzi pa tsiku kwa chaka)

[ngati ayankha kuti eya, funsani funso nambala 20A mpakana 20D ngati ayi pitani ku funso 20.2]

Kodi munali ndi zaka zingati nthawi imene mumayamba kusuta
_____ ____

<u>Ngati munasiya kusuta,</u> kodi mwasiya kusuta muli ndi zaka zingati?
ngati wotenga nawo mbali kusutabe lembani kuti '999'   _____ _____

C. Kodi mumasuta fodya mungati patsiku kapena
Pamulungu.?   i)____ ____ ____ ndudu pa tsiku
ii)___ ____ ____ ndudu pamulungu

Kodi mumasuta fodya wopangidwa ku kampani
kapena fodya wopichira nokha,   Wopangidwa ku kampani ☐

wopichira ☐

20.4. Kodi <u>munayambapo</u> mwasuta fodya wamu kaliwo? Eya ☐
Ayi ☐

("eya", akutanthauza kuti ansutapo ma ounce opyolera khumi ndi awiri a pipe tobacco mu moyo wawo)

[ngati ayankha kuti eya, funsani mafunso kuyambila funso namala 20.4A mpakana 20.4C ; ngati ati ayi pitani ku funso nambala 20.5]

Kodi munali ndi zaka zingati pamene munayamba kusuta pafupi pafupi fodya wa mu Kaliwo _____ _____

<u>Ngati munasiya kusuta,</u> kodi munasiya muli ndi zaka zingati? (ngati wotenga nawo mbali sanasiye kusuta lembaniponi kuti '999') _____ _____

Kwa nthawi yonse imene mwakhala mukusuta kapena imene munkasuta, kodi mumasuta mapipe angati patsiku kapena pa mulungu? (wonani pa ndondomeko wa mafunso A MRC) i)____ ____ ____ grams/pa tsiku
ii)____ ____ ____grams/pa mulungu

20.5. Kodi <u>munayambapo</u> mwasuta cigars, cheroots, or cigarillos?
Eya ☐
Ayi ☐

("Eya", akutanthauza kuti ndudu yopyolera imodzi ya cigar /ya cheroots / ya cigarillos pa mulungu kwa chaka mu muyo wawo)

[ngati ayankha kuti eya, funsani mafunso nambala 20.5A mapakana 20.5 C; ngati ati ayi, pitani ku funso nambala 20.6]]

A. Kodi munayamba kusuta fodya wa cigar/cheroot/cigarillos muli ndi zaka zingati? _____ _____

B. Ngati munasiya kusuta fodya, kodi munali ndi zaka zingati m'mene munkasiya? (ngatiwotenga nawo mbali sadasiye, lembaniponi kuti '999') ______ ____

C. Kwa nthawiyonse imene mwakhala mukusuta kapena imene munkasuta kodi mwasutapo

ndudu zingati pa tsiku kapena pa mulunngu? i) __ ___ ___ cigars etc/pa tsiku
ii)__ __ __cigars etc/pa mulungu

Note: Mutha kufinsa mafiso: 20.7 ndi 20.8 kapena ayi

20.7. munayambapo mwasutapo fodya wa chamba? Eya ☐
Ayi ☐

("eya," akutantawuaza kusuta majoints wo pyolela makumi awiri m'moyo wawo kapena joint imodzi pa mwezi kwa chaka nthawi inam iliyonse m'moyo wawo)

[ngati ayankha kuti eya funsani mafunso kuyambila funso nambala 20.8A mpakana 20.8C; ngati ati ayi pitani ku funso nmabala20.8)

Kodi munali ndi zaka zingati pamene munayamba
kusuta pafupipafupi fodya wa chamba? _____ _____

Ngati munasiya kusuta, kodi munasiya kusuta muli
ndi zaka zingati? (ngtiwotenganawo mbali sanasiye
lembaniponikuti '999') _____ _____

Nthawiyonse imene mwakhala mukusuta kapena
imene munkasuta, kodi mumasuta ndudu zingati
za chamba patsiku kapena pa mulungu? i)___ ____ ___ joints/pa tsiku
ii)___ ___ ___ joints/pa mulungu

20.8.Kodi munayambapo mwasutapo kapena kupumira mkati zinthu
Zokhala ngati fodya? Eya ☐
Ayi ☐
20.8.1. Mtundu ____
20.8.2. Mulingo/Kuchuluka _______________________________

[ngatiayankha kuti eya, funsani mafunso kuyambila funso nambala 20.8A mpakana 20.8C; ngati ati ayi, pitani kufunso nambala 21)

A.Kodi munali ndi zaka zingati pamene munayamba __ __
(________) kusuta?

B. Ngati mudasiya kusuta, kodi mudasiya muli ndi zaka zingati? (ngati

wotenga nawo mbalisadasiye lembaniponi kuti ‘999’) _____ _____

Kwa nthawi imene mwakhala mukusuta kapena imene munkasuta mumasuta fodya ochuluka bwanji angati pa tsiku kapena pa mulungu? i)__ __ __ unit/pa tsiku

ii)__ __ __unit/pa mulungu

[ngatiwotenga nawo mbali akusutabe fodya (funso nambala 20B palembedwa kuti ‘999,) ndiyeno funsani funso nambala 21A ndi 21B, ngati ati ayi pitani ku funso nambala 23]

21A. Mu chaka chapitachi, kodi mwasiyapo kusuta kangati kwa tsiku limodzi ____ ____ ____

21B. Kodi muli ndi malingaliro osiya kusuta pakadali pano? Eya, mkatikati mwa masiku makumi atatu akudzawa. ☐

Eya, mkatikati mwa miyezi isanu ndi umodzi ikudzayi. ☐

Ayi, sindikuganizirako zosiya kusuta. ☐

[ngati wotenganawo mbali sanasutepo fodya (wayankha kuti ayi ku ma funso ans kuyambila nambala 20.1 mapkana 20.5), ndiyepitaniku funso nambala 24.1. ngati ati eya pitilizani ndifunso nambala 23] pakadali pano palibepo funso nambala 22.

23. Kodi adotolo kapena a zaumoyo adakulangizaniponi kuti musiye kusuta fodya? Eya ☐

Ayi ☐

[ngati ayankha kuti eya, fusani funso nambala 23Andi 23B, ngati ati ayi, pitani ku funso nambala 24]

23A. Kodi mwalangizidwapo kuti musiye kusuta fodya mu miyezi khumi ndi iwiri yapitayi? Eya ☐

Ayi ☐

23B. Kodi mwagwiritsapo ntchito mankhwala ena aliwonse (Motsogoleledwa ndi adokotala kapena ayi) kuphatikizapo Makhwala omata pakhungu a nikotini kuti musiye kusuta? Eya ☐

Ayi ☐

[ngati ayankha kutimeya, funsani funso nambala 23B1, kenako funsani funso nambala 24. Ngati ati ayi pitani ku funo nambala 24]

23B1.Kodi ndi mtundu wanji wa mnkhwala umene

Munamwa kuti ukuthandizeni kusiya kusuta
Fodya? Nicotine Replacement ☐
Buproprion ☐
Tofranil ☐

Mankhwala ena ☐

24. Kodi mwagwiritsapo kapena kuchita china chilichonse
Kuti musiye kusuta fodya? Eya ☐
Ayi ☐

[ngati ayankha kuti eya, funsani funso nambala 24A, ngati ati ayi, pitani ku funso nambala 24.1]

24A. Kodi munapanga chani? Hypnosis ☐
Acupuncture ☐
Biofeedback ☐
Njira ina ☐

24.1. Osaziwerengelapo inuyo, kodi ndi anthu angati m'nyumba mwanu amene amasuta fodya pafupipafupi. __ __

24.2. Kodi anthu amsuta pafupipafupi fodya mu chipinda ku malo anu ogwirako ntchito? Yes ☐ No ☐
sagwira ntchito ☐

24.3. Kodi ndi ma ola angati patsiku amene mumayandikilana ndi anthu ena osuta fodya ku malo ngati awa?

24.3.1. ku nyumba ___ __

24.3.2. ku malo agwra ntchito ___ __
24.3.3. ku malo omwera mowa, odyera zakudya Owonwera kanema wa zinthunzi, kapena malo osangalalako akapangidwe koterowo ___ __
24.3.4. malo ena aliwonse ___ __

24.4. Kodi abambo anu anayambapo asuta fodya pafupipafupi nthawi yomwe munali mwana?
Eya ☐
Ayi ☐

24.5. kodi amayi anu anayambopo asuta fodya nthawi ya utsikana wawo ? Eya ☐
Ayi ☐

Chidziwitso, kakhalidwe ndinjira zomwe tingazindikile msanga za kusintha mukuwona ndikunva

Chiyambi: mafunso otsatirawa ndi okhudzana ndi ndudu ndi fodya

24.6. Kutengera pa zomwe mukudziwa kapena zomwe mumakhulupilira,Kodi kusuta fodya kukapangitse munthu kudwala kwambiri Eya ☐☐
Ayi ☐

[ngati ayankhakuti eya, funsani funso nambala 24.7, ngati ati ayi pitani ku funso nambala 25]

24.7. Kutengera pa zomwe mukudziwa kapena mumakhulupilira kodi kusuta fodya kungapangitse………

Werengani chinthu chinachilichonse	EYA	AYI	SINDIKUDZIWA
nthenda yo kufa ziwalo(magazi oundan na a muobongo amene amapangitsa kufa kwa ziwalo?	❑	❑	❑
b. Nthenda ya mtima?..................................	❑	❑	❑
c. Khasa ya mapapo?..................................	❑	❑	❑
d. Chifuwa cha mg'onagona?.........................	❑	❑	❑
e. Matenda mg'onagonokhuzana ndi mapapo. ntchito	❑	❑	❑

25. Kodi munagwirapo ntchito malo afumbi kwa chaka kapena kupyolera? Eya ☐

Ayi ☐

[ngati ayankha kuti eya, funsani funso nambala 25A nagti ati ayi, itani ku funso nambala 26]

25A. Mwakhala kwa zaka zingati mukugwira malo a fumbi? ____________

Zinthu zina zingaonjezere kusapeza bwino

26. Kodi adotolo kapena ena aliwonse a za umoyo adakuwuzanipo kuti muli ndi nthenda ya :

26A. Mtima Eya ☐
Ayi ☐

26A. Kulephera kugwira ntchito kwa mtima
Eya ☐
Ayi ☐

26B. Vuto lothamanga magazi Eya ☐
Ayi ☐

26C. Matenda a shuga Eya ☐
Ayi ☐

26D. Khansa ya mapapo Eya ☐
Ayi ☐

26E. Kufa ziwalo Eya ☐
Ayi ☐

26F. Chifuwa chachikulu Eya ☐
Ayi ☐

[ngati ayankha kuti eya, ku funso nambala 26F ndiye funsani funso nambala 26F1; ngati ati ayi, pitani ku funso nambala 27]]

26F1. Kodi mukumwa mankhwala a chifuwa cha chikulu? Eya ☐
Ayi ☐
[ngati ayankha kuti ayi ku funso nambala 26F1 , ndiye funsani funso nambala 26F2; ngati akuti eya pitani ku funso nambala 27]

26F2. Kodi munayambapo mamwa mankhwala a chifuwa chachkulu? Eya ☐

Ayi ☐

27. Kodi munayambapo mwa pangidwa opareshoni pa chifuwa imene inachititsa kuchotsapo mbali imodzi la phapo lanu?Eya ☐
Ayi ☐

28. Kodi munayambapo mutagonekedwa m'nchipatala ndi vuto lokanika kupuma <u>musanakwanitse</u> zaka khumi? Eya ☐
Ayi ☐
sindikudziwa ☐

29. Mu miyezi khumi ndi iwiri yapitayi, munalandilapo katemera wa chimfine? Eya ☐
Ayi ☐
sindikudziwa ☐

30. Kodi adotolo kapena azaumoyo adawauzapo bambo anu, mayi anu, chemwali wanu kapena nchimwene wanu kuti ali

ndi nthenda ya m'mapapo yomwe imapangitsa kubanika,
chifuwa cha m'gona gona kapena matenda otseka njira
yo pumira (COPD)? Eya ☐
Ayi ☐

31. kodi pali anthu ena a nyumba mwanu (kupatulapo inuyo)
asutapo fodya wandudu kaliwo kapena
chi ndudu chachikulu musabata ziwiri zapitazi? Eya ☐
Ayi ☐

SF12

Ofunsa mafunso: werengani malangizo kwa munthu ofunsidwa.
Malangizo: kafukufukuyu ndi ofuna kudziwa mmene thanzi lanu lilili. Izi zithandiza ku londoloza za mmene inuyo mumanvera nthupi mwanu ndiponso mmene mumagwirila ntchito zanu za tsiku ndi tsiku. Pa funso linalilonse chonde sankhani funso lomwe mukuliwona kuti likukamba bwino za inuyo.

32. Munganenepo chani za umoyo wanu : *(Check one)* labwino kwa mbiri zedi ☐

La bwino kwa mbiri ☐
Labwino ☐
lilibwinoko ☐
sililibwino ☐

33. Mafunso ali munsimu ndi okhudzana ndi zomwe mungachite tsiku ndi tsiku. Kodi nthanzi
lanu panopa limakulepheretsani kugwira ntchitozi? Ngati zili choncho, zimakulepheretsani bwanji?

33A. *Ntchito zopepukirapo*, ngati kusuntha gome
Kukolopa kusewera masewero a mpira kapena kusewera
Ntchito za kudimba. eya, ndimakanika kwambiri ☐

Eya, ndimakanika pang'ono ☐
sindimakanika ☐

33B. Kukwera ma sitepe angapo? Eya,Ndimakanika kwambiri ☐

Eya Ndimakanika pang'ono ☐

Sindimakanika ☐

34. Kodi mu milungu inayi yapitayi, kodi ndikangati komwe mwakhala Ndi mavuto awa ndi ntchito yanu kapena mu ntchito zanu zina chifukwa cha umoyo wanu?

34A. Mumakwaniritsa mochepa kuyelekeza ndi monga Mmene mumayembekezera

- nthawi zonse ☐
- Nthawi zambiri ☐
- Nthawi zina ☐
- Nthawi pango'ono ☐
- palibe ☐

34B. Mumagwira ntchito kapena kupanga zinthu zina osati momwe mumapangira nthawi zonse

- nthawi zonse ☐
- Nthawi zambiri ☐
- Nthawi zina ☐
- Nthawi pango'ono ☐
- palibe ☐

35. Kwa milungu inayi yathayi, kodi ndi kochuluka bwanji komwe mwakhala ndi chimodzi chan mavuto ndi ntchito yanu kapena ntchito zanu za tsiku ndi tsiku kaamba ka vuto la maganizo?(monga ngati kukhumudwa)?

35A. Mumakwaniritsa mochepa kuyelekeza ndi monga Mmene mumayembekezera

- nthawi zonse ☐
- Nthawi zambiri ☐
- Nthawi zina ☐
- Nthawi pango'ono ☐
- palibe ☐

35B.Mumagwira ntchito kapena kupanga zinthu zina osati momwe mumapangira nthawi zonse?

- nthawi zonse ☐
- Nthawi zambiri ☐
- Nthawi zina ☐
- Nthawi pango'ono ☐
- palibe ☐

36. Kwa milungu inayi yapitayi, kodi ndikangati komwe ululu wanu wakulepheretsai kugwira ntchito zanu za tsiku ndi tsiku

palibe nkomwe ☐
pang'ono ☐
Mochulikirapo ☐
Mochuluka kwambiri ☐
Mochuluka kwambiri zedi ☐

37. Mafunsowa ndi okhudzana ndi m'mene mukunvera mthupi mwanu ndim'mene zinalili ndi inuyo pa milungu inayi yathayi. Pa funso linalilonse, chonde perekani yankho limodzi lomwe likulongosola moyenerera m'mene mwakhala mukunvera.

Kodi ndikangati pa milungu inayi yomwe ya pitayi….

37A. Momwe nthupi mwanu munamva bwino bwino?

nthawi zonse ☐
Nthawi zambiri ☐
Nthawi zina ☐
Nthawi pango'ono ☐
palibe ☐

37B. Mumakhla ndi mphanvu zambiri?

nthawi zonse ☐
Nthawi zambiri ☐
Nthawi zina ☐
Nthawi pango'ono ☐
palibe ☐

37C. Munakhala okhumudwa?

nthawi zonse ☐
Nthawi zambiri ☐
Nthawi zina ☐
Nthawi pango'ono ☐
palibe ☐

38. Pa milungu inayi yapitayi, kodi kochuluka bwanji komwe Moyo wanuwo kapena vuto la maganizo anu lakulepheretsani kugwira ntchito kapena kuchita zochita zanu za tsiku ndi tsiku? (monga kuyendera anzanu, abale anu, ndi zina zotero) nthawi zonse ☐

Nthawi zambiri ☐

Nthawi zina ☐

Nthawi pango'ono ☐

palibe ☐

Copyright © XXXX Medical Outcomes Trust. All rights reserved. (SF-12 Standard U.S. Version 2.2)

Zokhudzana ndi zachuma

Nthawi ya ntchito yomwe inatayika

Mafunso otsatirawa ndi okhudzana ndi ntchito imene mumagwira komanso masiku amene mwajomba chifukwa cha vuto la umoyo wanu

39. Mwa miyezi khumi ndi iwiri yapitayi mwagwirapo ntchito yolipidwa? Eya ☐

Ayi ☐

[ngati ayi, pitilizani ndi funso nambla 39A; ngati eya, pitani kufunso nambala 44]

39A. Mwa miyezi khumi ndi iwiri yathayi, mwakanika kugwira ntchito yolipidwa Chifukwa cha vuto la mapumidwe? Eya ☐
Ayi ☐

39B. Mwa miyezi khunmi ndi iwiri yapitayi,simunagwire ntchito yolipidwa chifukwa munali wokhala pakhomo kapena mumasamalira wina? Eya ☐

Ayi ☐

[ngati eya, pitilizani ndi funso nambala 39C, ngati ayi, pitani ku funso nambala 40]

39C. Mwa miyezi khumi ndi iwiri yapitayi, kodi vuto la umoyo wanu linakulepheretsani ntchito zanu za pakhomo za masiku onse kapena kusamalira munthu wina wake? Eya ☐
Ayi ☐

[ngati eya, pitilizani ndi funso nambala 39D ndi funso nambala 39E, ngati ayi, pitani ku funso nambala44]

39D. Mu miyezi khumi ndi iwiri yathayi kodi ndi masiku angati omwe Mwalephera kupanga ntchito zanu zapakhomo kapena Kusamalira wina kaamba ka vuto la umoyo wanu? ____ ____ ____

39E. Mu miyezi khumi ndi iwiri yapitayi, kodi ndi masiku angati omwe mwalephera kugwira kapena kuchito ntchito zanu za pakhomo kapena kusamalira wina kaamba kweni kweni ka vuto la mapumidwe____ ____ ____

[chonde pitani ku funso nambala 44]

40. Kodi mwagwira ntchito yolipidwa kwa miyezi ingati mu miyezi khumi ndi iwiri yathayi? _____ _____

41. Munthawi imene mumagwirayi ntchito yolipidwayi, kodi ndi masiku angati pa Mulungu amene mumagwira ntchito? ____ _____

42.Kodi mumagwira ntchito yolipidwayi kwa maola angati pa tsiku? _____ _____

43.Mu miyezi khumi ndi iwiri yathayi, kodi vuto la umuyo wanu linakulepheretsani kugwira ntchito yolipidwayi? Eya ☐
Ayi ☐

[ngti eya, pitilizani ndi funso nambala 43A ndi 43B, ngati ayi, pitani ku fiunso nambala 44]

43A. Mu miyezi khumi ndi iwiri yathayi, kodi ndi masiku okwana angati Munalephera kugwira ntchito yolipidwa kaamba ka vuto la umoyo wanu? ____ ____ ____ days

43B. Mumiyezi khumi ndi iwiri yathayi, kwa masiku angati Amene munalephera kugwira ntchito yolipidwa kwenikweni kaamba ka vuto la mapumidwe? ____ ____ ____ days

zinthu zina zosakhudzana ndi ntchito zomwesizinakwanilitsidwe

Mafunso otsatirawa ndi okhudzana ndi nthawi yomwe yataidwa posakwanitsa ntchito zanu za tsiku ndi tsiku (monga kukagula zinthu, kuyendela anzanu/abale, kupita ku tchalichi kapena zinthu zina) chifukwa cha mavuto a umoyo wanu

44. Mu miyezi khumi ndi iwiri yathayi, kodi mavuto a umoyo wanu anakulepheretsani kutenga nawo mbali pa zochitika kamodzi kapena kawiri kawiri? Eya ☐
Ayi ☐

[ngati eya, yankhani funso nambala 44A ndi 44B, ngati ayi, pitani ku mawu olemba kuti yamalizidwa ndi kumapeto kwa ndondomeko ya mafunsowa]

44A. Mu miyezi khumi ndi iwiri yapitayi, ndi kwa masiku angati amene simunatengepo mbali mu zinthu zochitika zosagwirizana ndi ntchito zanu kaaba ka vuto la umoyo wanu? ____ ____ ____ days

44B. Mumiyezi khumi ndi iwiri yapitayi, kwa masiku angati amene simunatenge nawo mbali pa zinthu zochitika kaamba kwenikweni ka vuto la mapumidwe? ____ ____ ____ days

yamalizidwa ndi: ____ ____ ____ ____

1.6 Appendix F: Chichewa translation of the Spirometry questionnaire.

TSAMBA LA NDONDOMEKO YA NTCHITO YOPHWEKA YOPEZA NDI KUYANG'ANILA MAVUTO A MAPAPO POGWIRITSA NTCHITO KUPUMA MU KAFUKUFUKU WA BOLD

Mafunso ofuna kukutetezani

1. Mu miyezi itatu yadutsayi mwapangidwapo opaleshoni ina iriyonse pachifuwa kapena pa mimba panu? Inde ☐ Ayi ☐

2. Kodi mwakhalapo ndi vuto la mtima mkati-kati mwa miyezi itatu yadutsayi? Inde ☐ Ayi ☐

3. Kodi muli ndi kachikopa kofewa kwambiri ka diso kapadela Kapena mwakhalapo ndi opaleshoni ya diso mkati-kati mwa miyezi Itatu yapitayi? Inde ☐ Ayi ☐

4. Kodi mwagonekedwapo mchipatala chifukwa cha vuto lina lirilonse la mtima mkati-kati mwa mwezi wapitawu? Inde ☐ Ayi ☐

5. Kodi muli gawo lachitatu la kukhala ndi pakati/ kwasala miyezi itatu kuti mu beleke? Inde ☐ Ayi ☐

6. Kodi otenga nawo mbariwa mtima wawo ukugunda kokwana mlingo 120 atangokhala akamapumula pa mphindi iriyonse? Inde ☐ Ayi ☐

7. Kodi pakali pano mukumwa mankhwala a TB? Inde ☐ Ayi ☐

8. *Kodi pali chifukwa china chimene otenga nawo mbari uyu Sakuyenera kupanga nawo ndondomeko ya ntchito yophweka yopeza ndi kuyang'anila mavuto a mapapo pogwiritsa ntchito kupuma?* Inde ☐ Ayi ☐

*ngati yankho ku ena aliwonse mwa mafunso 1 kudutsa 8 liri "Inde", **MUSA** pitilize ndi ntchito yoyezayi mapapo. Pitani ku gawo la zotsatira pomwe pali ntchito yophweka yopeza ndi kuyang'anila mavuto a mapapo pogwiritsa ntchito kupuma ndipo ikani chizindikiro pa mafunso 11A ndi 11B choti "Ayi", ndipo onani bokosi yachiwiri "otenga nawo mbari wachotsedwa pa nkhani ya mndandanda wa mankhwala", ku funso 11C.*

9. Kodi mwakhalapo ndi matenda okhudzana ndi kupuma (chinfine) mu masabata atatu adutsawa? Inde ☐ Ayi ☐

10.1. Kodi mwamwapo mankhwala ena aliwonse pa vuto la mapumidwe mu maola 24 apitawa? Inde☐

Ayi ☐

*Ngati ali **Inde**, lembani dzina/mtundu wa mankhwala agwiritsidwa ntchito.*

____

____

____

Ngati funso 10.1 liri Inde ndipo mankhwala agwiritsidwa ntchito akuphatikizapo ina iriyonse mwa mitundu yotsatira m'munsimu, pitani ku funso 10.2.ngati ali Ayi,pitani ku funso 10.5.

MTUNDU WA MANKHWALA	ZITSANZO	
Short-acting beta-2 agonist	albuterol, salbutamol	Maola 6 ife tisanafike panyumba pano
Anticholinergic inhaler	Atrovent, ipratropium	Maola 6 ife tisanafike panyumba pano
Long-acting beta-2 agonist (kuphatikizapo zosakaniza zokonzedwa zimene ziri ndi LABA)	Serevent, Advair, formoterol, Symbicort	Maola 12 ife tisanafike panyumba pano
Oral beta-2 agonist	Albuterol	Maola 12 ife tisanafike panyumba pano
Oral theophylline	Theodur	Maola 12-24 ife tisanafike panyumba pano.
Long-acting anticholinergic	Spiriva, tiotropium	Maola 24 ife tisanafike panyumba pano

10.2. *kodi otenga nawo mbari anagwiritsa ntchito **short-acting beta-2 agonist** kapena **anticholinergic inhaler,** kaya paokha kapena pophatikiza zinthu zina,mu maola asanu ndi chimodzi apitawa?* Inde ☐

Ayi☐

10.3 *kodi otenga nawo mbari anagwiritsapo ntchito long-acting beta-2 agonist kapena ndi zinthu zina,mu maola 12 adutsawa?* Inde☐

Ayi☐

10.4. *kodi otenga nawo mbari anagwiritsilapo ntchito **Oral theophylline*** / **long acting anticholinergic***, Kaya payokha kapena kuphatikiza zinthu zina/mankhwal, M'maola 24 apitawa?* Inde ☐

☐☐☐☐☐☐☐☐☐☐☐☐☐☐☐☐☐☐☐☐☐☐☐☐☐☐☐☐☐☐Ayi ☐

10.5.Kodi mwasuta komaliza liti? i) maola____ ____ ____apitawa
ii) masiku____ ____apitawa

lembani 999 ngati ali osasuta kapena osiya kusuta (sanasutepo mu mwezi umodzi wapitawo)

10.7. Kugunda **bpm ___ ____ ____**

10.8. Kutalika **____ ____ ____cm**

10.9 Kulemela **____ ____ ____.____kg**

10.10.A. Muyezo oyamba wa mu malekezelo a ntchafu: **____ ____ ____.____cm**

10.10.B. Muyezo wachiwiri wa mu malekezelo a ntchafu **____ ____ ____.____cm**

10.11.A. Muyezo oyamba wa mu malekezelo a chiuno **____ ____ ____.____cm**

10.11.B. Muyezo wachiwiri wa mu malekezelo a chiuno **____ ____ ____.____cm**

Spirometry Outcome

11A.	Acceptable pre-bronchodilator test completed?	Yes ☐<br>No ☐
11B.	Acceptable post-bronchodilator test completed?	Yes ☐<br>No ☐
11C.	Unable to obtain satisfactory spirometry (check one)	
	The participant did not understand instructions	☐
	The participant was medically excluded	☐
	The participant was unable to physically cooperate	☐
	The participant refused	☐

12. Kodi zinakuchitikirani zoipa zina zirizonse zokhudzana ndi ndondomeko ya ntchito yophweka yopeza ndi kuyang'anila mavuto amapapo pogwiritsa ntchito kupuma pa nthawi yoyeza? Inde ☐
Ayi ☐

Ngati Inde,chonde fotokozani mwa chidule za chochitika chofunikiracho:

____

____

____

____

13. Ngati otenga nawo mbari anali ndi chochitika choti chikhoza kukhuza zotsatila za ntchito yao yophweka   yoyeza ndi kuyang'anila mavuto a mapapo pogwiritsa ntchito kupuma (mwachitsanzo, kyphosis, ziwalo zosowa, etc.) Lembani chochitika apa.

14. Nambala ya ogwira ntchito m'madela

___ ___ ___ ___
